# Welfare implications for broiler chickens reared in an insect larvae-enriched environment: Focus on bird behaviour, plumage status, leg health, and excreta corticosterone

**DOI:** 10.3389/fphys.2022.930158

**Published:** 2022-08-25

**Authors:** Ilaria Biasato, Sara Bellezza Oddon, Giulia Chemello, Marta Gariglio, Edoardo Fiorilla, Sihem Dabbou, Miha Pipan, Dominik Dekleva, Elisabetta Macchi, Laura Gasco, Achille Schiavone

**Affiliations:** ^1^ Department of Agricultural, Forest and Food Sciences, University of Turin, Turin, Italy; ^2^ Department of Life and Environmental Sciences, Marche Polytechnic University, Ancona, Italy; ^3^ Department of Veterinary Sciences, University of Turin, Turin, Italy; ^4^ Center Agriculture Food Environment, University of Trento, Trento, Italy; ^5^ Entomics Biosystems, Cambridge, United Kingdom

**Keywords:** black soldier fly, broiler chickens, environmental enrichment, welfare, yellow mealworm

## Abstract

The use of insect live larvae as environmental enrichment has recently been proposed in broiler chickens, but the concomitant administration of black soldier fly (BSF) and yellow mealworm (YM) has never been tested yet. Therefore, the present study aims to evaluate the effects of live BSF and YM larvae as environmental enrichments for broiler chickens by means of plumage status, behaviour, leg health, and excreta corticosterone metabolites (CM). A total of 180 4-day old male Ross 308 broiler chickens were randomly distributed in 3 experimental treatments (6 replicates/treatment, 10 birds/replicate) and fed for 35 days as follows: 1) control (C, commercial feed), 2) BSF: C + 5% of the expected daily feed intake [DFI] live BSF larvae and 3) YM: C + 5% of the expected DFI live YM larvae. Feathering, hock burn (HB) and footpad dermatitis (FPD) scores (end of the trial), as well as behavioural observations (beginning of the trial [T0] and every 11 days [T1, T2 and T3] during morning, larvae intake and afternoon) through video recordings, were assessed, and excreta samples collected to evaluate the CM. Feathering, HB and FPD scores, and excreta CM were unaffected by insect live larvae administration (*p* > 0.05). In the morning, the insect-fed birds displayed higher stretching, wing flapping, ground pecking (at T1 and T3), as well as lower preening (at T1 and T2), than the C group (*p* < 0.05). During the larvae intake, higher scratching, wing flapping and ground pecking, as well as lower stretching, preening and laying down, were observed in the insect-fed (scratching, stretching and laying down) or YM-fed (wing flapping, ground pecking and preening) groups than the C birds (*p* < 0.05). In the afternoon, insect live larvae administration increased wing flapping (YM) and laying down (BSF and YM), as well as decreased ground pecking (YM, *p* < 0.05). In conclusion, the administration of insect live larvae as environmental enrichment (especially YM) was capable of positively influencing the bird welfare through the stimulation of foraging behaviour, increase in activity levels, and reduction in bird frustration, without affecting the plumage status, leg health, and excreta CM.

## Introduction

Insects are nowadays recognized as excellent biofactories for their peculiar ability to valorise a wide spectrum of waste materials by nutrition upcycling, which allows obtaining edible high-quality micro- and macro-nutrients that can be incorporated in the animal feed chain (Gasco et al., 2020). The so-obtained insect larvae are, indeed, predominantly fractionated to obtain meals and oils, which can efficiently be utilized to replace the conventional protein and lipid sources in monogastric diets (Ravi et al., 2020). However, the scientific research recently carried on revealed that insect live larvae may also potentially reach an interesting market share in the form of environmental enrichments for either poultry (Pichova et al., 2016; Veldkamp and van Niekerk, 2019; Ipema et al., 2020; Star et al., 2020; Bellezza Oddon et al., 2021; Tahamtani et al., 2021) or pigs (Ipema et al., 2021a; Ipema et al., 2021b).

Environmental enrichment can be defined as a modification of the rearing environment of captive animals aimed at improving their biological functioning and stimulating their species-specific behaviours (Newberry, 1995). The enrichment strategies currently available for broiler chickens can be grouped in 2 main categories: 1) “point-source objects”, which are enrichment objects/devices that are generally limited in size and whose use is often restricted to a single or a few locations in an animal enclosure; and 2) more complex enriched environments designed to meet the key behavioural needs of the animals within them (i.e., outdoor access) (Riber et al., 2018). Among the “point-source objects”, the provision of food items to stimulate the bird foraging activity represents one of the most practical and effective enrichment techniques, as search for various types of food resources on the litter has been reported to increase foraging and movement in broiler chickens (Pichova et al., 2016; Ipema et al., 2020). Such increase in overall activity levels may have implications for the intensive farming, where the fast growth rates and the high body weights are the main cause of leg problems and lameness in broilers, thus, in turn, deeply limiting their ability to move (Reiter and Bessei, 2009). Furthermore, as fast-growing broilers spend between 60 and 80% of their time sitting (de Jong and Gunnink, 2018), contact dermatitis (i.e., hock burns, breast burns and foot pad dermatitis) may also frequently occur, as a consequence of continuing contact and pressure of the skin of the breast, hocks and feet against humid and soiled bedding (Ekstrand et al., 1998). The limited space and the absence of environmental stimuli of the commercial conditions can also impair broiler welfare by limiting the possibility to perform intrinsically motivated behaviours and diminishing activity levels, thus, in turn, furtherly increasing the occurrence of leg problems (Vasdal et al., 2019), and the susceptibility to abdominal dermatitis, plumage soiling and feet and hock dermatitis (Bruce et al., 1990; Opengart et al., 2018).

Black soldier fly (BSF) and yellow mealworm (YM) live larvae provision has recently been proposed as promising food environmental enrichment to promote welfare in broiler chickens, with increased activity and foraging behaviour (as a result of the search for larvae on the ground), and reduced occurrence of hock burns and lameness (as a result of the increased activity) being observed in the administered birds (Pichova et al., 2016; Ipema et al., 2020). Welfare assessment in broiler chickens is usually object of a multiperspective approach, as heterogeneous parameters (such as plumage status, hock burns and footpad dermatitis, lameness, behavioural patterns, and excreta corticosterone) are commonly evaluated (Weimer et al., 2018; Giersberg et al., 2021; Iannetti et al., 2021; Lourenço da Silva et al., 2021). Despite beneficial live insect larvae-related effects on bird behaviour and feathering scores having recently been highlighted in either turkeys (Veldkamp and van Niekerk, 2019) or laying hens (Star et al., 2020; Tahamtani et al., 2021), data about modulation of plumage status and excreta corticosterone in broiler chickens reared in live insect larvae-enriched environment are still missing. Furthermore, no studies assessing the effects of the concomitant administration of BSF and YM live larvae as environmental enrichments are currently available in poultry.

Therefore, the present study aims to investigate the effects of BSF and YM live larvae as environmental enrichments for broiler chickens, assessing the implications for bird welfare by means of behaviour, plumage status, leg health, and excreta corticosterone metabolites (CM).

## Materials and methods

### Birds and experimental design

The experimental design of the present study is reported in details by Bellezza Oddon et al. (2021), as the current research is part of the same project and was performed using the same birds. In order to provide a brief summary, a total of 180 4-day old male Ross 308 broiler chickens were randomly allotted to 3 experimental treatments (6 replicate pens/treatment, 10 birds/treatment) as follows: 1) control (C), where a commercial feed only was provided (two feeding phases: starter [4–11 days] and grower-finisher [12–38 days]; ii), BSF, where the C diet was supplemented with 5% of the expected daily feed intake [DFI] of BSF live larvae (calculated on dry matter [DM]); and 3) YM, where the C diet was supplemented with 5% of the expected DFI of YM live larvae (DM). The starter commercial feed was characterized by 12.5 MJ/kg metabolizable energy (ME) and 224 g/kg crude protein (CP), while the grower feed contained 13.0 MJ/kg ME and 220 g/kg CP (Fa.ma.ar.co SPA, Cuneo, Italy). The pens were 1.20 m wide × 2.20 m long (bird density at the end of the growth: 10 kg/m^2^). The daily amount of live larvae was distributed to all the pens in two plates at the same hour (11.00 a.m.) and 7 days/week for the whole trial (35 days). To avoid any potential bias, two plates with a known amount of control feed inside were also provided to the C animals to create the same interaction with the operators in all the treatments, and there was also a visual separation among the pens (Bellezza Oddon et al., 2021).

### Feathering score

At the end of the experimental trial, all the birds were given feathering scores for back, breast, wing, under-wing and tail using scores of 1–5 for feather coverage as follows: score 1, minimal coverage (<25% coverage); score 2, 25%–50% coverage; score 3, 50%–75% coverage; score 4, >75% coverage; and score 5, complete coverage (Lai et al., 2010).

### Behavioural observations

The behavioural observations were carried out using video recordings. A total of 3 pens/treatment were filmed for 5 min in the morning (9.00–9.05 a.m.), 5 min during the larvae intake (11.00–11.05 a.m.) and 5 min in the afternoon (6.00–6.05 p.m.) at the beginning of the trial (T0) and every 11 days until the end of the experiment (T1, T2 and T3). The recorded videos were analysed by the Behavioural Observation Research Interactive Software (BORIS, v 7.9.7) (Friard and Gamba, 2016). The considered behaviours were divided in two categories: the frequency (point event) and the duration (state event) behaviours (Table 1). The frequency behaviours were evaluated as the number of times that a specific behaviour occurred in the pen during the 5 min periods of observations. The duration behaviours were, instead, assessed as the percentage of the 5 min periods of observations that 4 identified subjects in the pen (named as alpha, beta, gamma and delta) spent performing a specific behaviour.

**TABLE 1:** Description of the broiler ethogram (frequency and duration behaviours) considered in the present study.

Frequency behaviour	Definition
Scratching	Scraping of the litter with the claws (Ipema et al., 2020)
Preening	Grooming of own feathers with beak (Ipema et al., 2020)
Trotting	Increasing walking step with head high and breast out (Veldkamp and van Niekerk, 2019)
Pecking pen mate	Pecking movements directed at the body or beak of a pen mate (Ipema et al., 2020)
Stretching	Stretching one wing together with the leg at the same side or both wings upward and forward (Martin et al., 2005)
Chasing	One hen chasing another, with fast running, no vocalisations, no hopping and no wing flapping (Sokołowicz et al., 2020)
Wing flapping	Number of wing beats, often while the bird is standing on the toes (Martin et al., 2005)
Shaking	Body/wing shake when the plumage is not in order (Martin et al., 2005)
Dust bathing	Sitting and performing: vertical wing-shaking, body shaking, litter pecking and/or scratching, bill raking, side and head rubbing (van Hierden et al., 2002)
Allopreening	Social preening (Kenny et al., 2017)
**Duration behaviours**	**Definition**
Walking	Taking one or more step (Webster and Hurnik, 1990)
Preening	Grooming of own feathers with beak (Ipema et al., 2020)
Standing still	Standing on the feet with extended legs (Webster and Hurnik, 1990)
Ground pecking	Pecking at the litter with the head in lower position than the rump (van Hierden et al., 2002)
Lying down	Sitting position (Webster and Hurnik, 1990)

### Feet and hock health assessment

The feet and hocks of the broiler chickens were examined at the end of the experimental trial in order to assess the incidence and the severity of the footpad dermatitis (FPD) and the hock burns (HB). The FPD was scored as follows: 0 = no lesion, slight discoloration of the skin or healed lesion; 1 = mild lesion, superficial discoloration of the skin and hyperkeratosis; and 2 = severe lesion, affected epidermis, blood scabs, haemorrhages and severe swelling of the skin (Ekstrand et al., 1998). Differently, the HB were scored as follows: 0 = no lesion; 1 = superficial, attached (single) lesion or several single superficial or deep lesions ≤0.5 cm; 2 = deep lesion >0.5 cm to ≤1 cm or superficial lesion >0.5 cm; 3 = deep lesion >1.0 cm; 4 = whole hock extensively altered (Louton et al., 2020).

### Excreta corticosterone analysis

At the beginning of the trial (T0) and every 11 days (after the video recordings of the administration of the insect live larvae) until the end of the experiment (T1, T2, and T3), all the birds from each pen were housed in wire-mesh cages (100 cm width × 50 cm length) for 120 min to collect fresh excreta samples. After collection, the excreta samples were pooled, immediately frozen at −20°C until corticosterone analysis, and processed according to Palme et al. (2013) and Costa et al. (2016). In particular, the excreta were freeze-dried and ground using a cutting mill (MLI 204; Bühler AG, Uzwil, Switzerland). A total of 0.25 g of the samples were placed into an extraction tube with 3 ml of ether and stored at −20°C for 1 h. After this time, the aliquots were mixed for 3 min through multivortex and the supernatant was recovered and transferred in a new tube. The tubes were then placed at 50°C for 14 h to obtain a dried extract. Lastly, excreta CM were analysed with a multi species enzyme immunoassay kit (Arbor Assay^®^, Ann Arbor, MI, United States) developed for serum, plasma, saliva, urine, extracted faecal samples, and tissue culture media. All of the analyses were performed in duplicate. The inter- and intra-assay coefficients of variation were less than 10% (7% and 9%, respectively). The sensitivity of the assay was 11.2 ng/g of excreta. All of the samples were analysed at multiple dilutions (1:4, 1:8, 1:16, and 1:32) and all the regression slopes were parallel to the standard curve (r^2^ = 0.979).

### Statistical analysis

The statistical analysis was performed using IBM SPSS Statistics V28.0.0 software (IBM, Armonk, NY, United States). The pen was considered as the experimental unit for the plumage status, behaviour, and excreta CM analyses, while the bird was used for the assessment of the leg health. Shapiro-Wilk’s test established normality or non-normality of distribution of both the data and the residuals. The feathering scores were analysed by fitting a generalized linear mixed model (GLMM) that allowed them to depend on linear predictors (diet, time, and their interaction) through a negative binomial response probability distribution with a nonlinear link function (log). The mean scores of each body area were included in the statistical model. A GLMM was also fit to allow the behaviour data to depend on the same linear predictors through a Poisson loglinear distribution (frequency behaviours) or a gamma probability distribution with a nonlinear (log) link function (duration behaviours). The total number of times that the specific frequency behaviours occurred in the pen, as well as the mean percentage of time that the 4 identified subjects of the pen spent performing the specific duration behaviours, were included in the corresponding statistical models. Frequency behaviours occurring less than 0.5 times on average per period of observation were excluded from the GLMM. The excreta CM were also analysed by fitting a GLMM that allowed them to depend on the same linear predictors through a gamma probability distribution with a nonlinear link function (log). The mean CM resulting from the duplicate analysis was included in the statistical model. The replicate was included as a random effect to account for repeated measurements on the same pen, and the interactions between the levels of the fixed factors were evaluated by means of pairwise contrasts. The HB and FPD scores were analysed by means of Kruskal-Wallis (post-hoc test: Dunn’s Multiple Comparisons Test). The results were expressed as least square mean (plumage status, behaviour, and excreta CM) or mean (leg health) and standard error of the mean (SEM). *p* values ≤0.05 were considered statistically significant.

## Results

### Feathering score

The feathering scores of the broiler chickens of the current research are summarized in Table 2. The administration of both the BSF and the YM live larvae did not influence the feathering scores of the birds (*p* = 0.545). On the contrary, the feathering scores depended on the body area (*p* < 0.001). In particular, the back showed better scores when compared to the other body areas, with breast, under-wing and tail furtherly displaying greater scores than the wing (*p* < 0.001). No diet × body area interaction was also identified (*p* = 0.237).

**TABLE 2:** Feathering score of the broiler chickens depending on diet, body area and their interaction.

	Diet (D)			Body area (B)					SEM		*p*-value			Wald test		
	C	BSF	YM	Back	Breast	Wing	Under-wing	Tail	D	B	D	B	D×B	D	B	D×B
Score, n	1.18	1.16	1.21	3.19^a^	1.00^b^	0.73^c^	1.00^b^	0.99^b^	0.03	0.05	0.545	<0.001	0.237	1.214	854.780	8.010

C = control group; BSF = C diet + black soldier fly live larvae; YM = C diet + yellow mealworm live larvae. Means with superscript letters (a, b, c) denote significant differences (*p* < 0.05).

### Behaviour analysis

**FIGURE 1:**
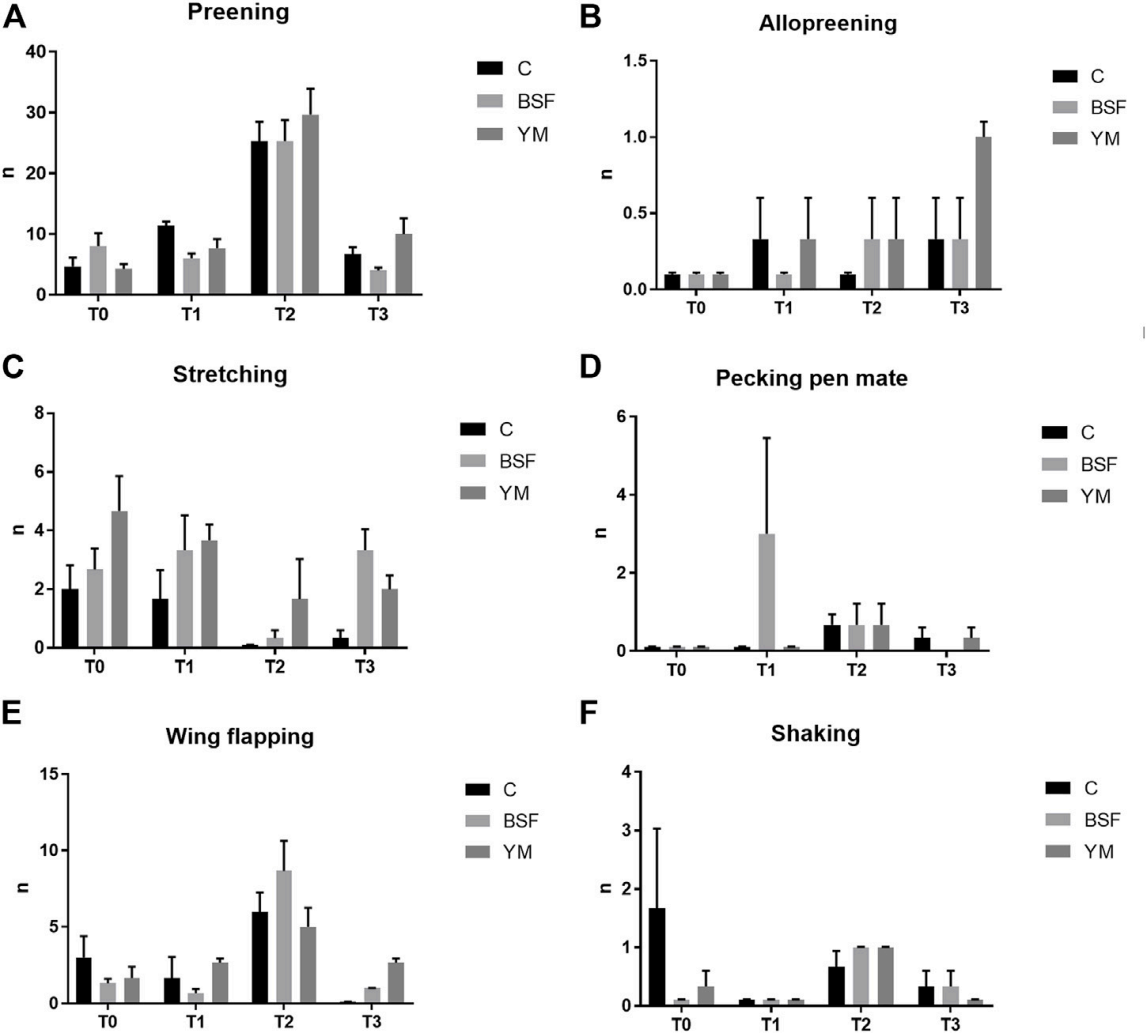
Frequency behaviours of the broiler chickens in the morning (diet*time interaction, *p* > 0.05). **(A)** Preening. **(B)** Allopreening. **(C)** Stretching. **(D)** Pecking pen mate. **(E)** Wing flapping. **(F)** Shaking. C = control group; BSF = C diet + black soldier fly live larvae; YM = C diet + yellow mealworm live larvae. T0 = day 0; T1 = day 11; T2 = day 22; T3 = day 33.

**FIGURE 2:**
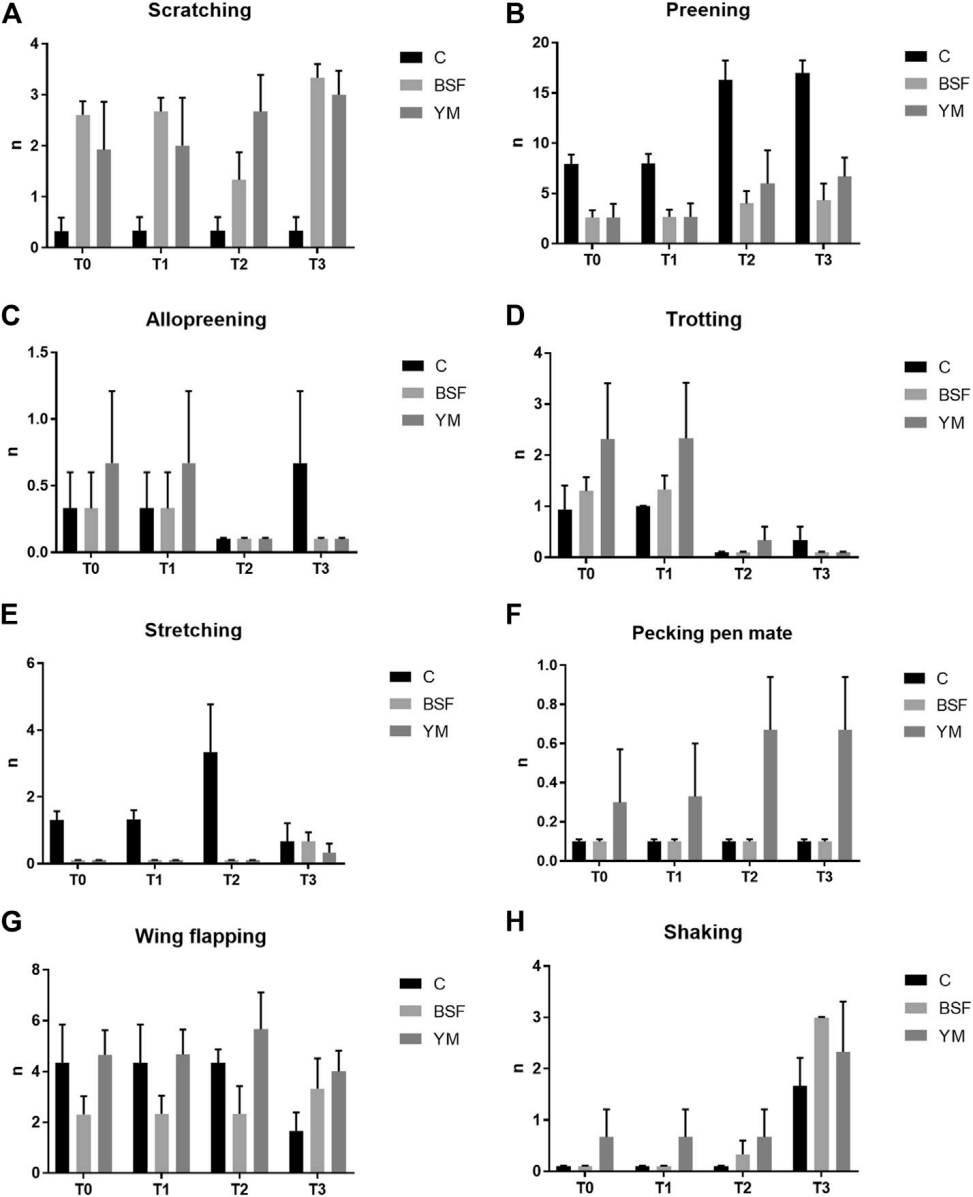
Frequency behaviours of the broiler chickens during the larvae intake (diet*time interaction, *p* > 0.05). **(A)** Scratching. **(B)** Preening. **(C)** Allopreening. **(D)** Trotting. **(E)** Stretching. **(F)** Pecking pen mate. **(G)** Wing flapping. **(H)** Shaking. C = control group; BSF = C diet + black soldier fly live larvae; YM = C diet + yellow mealworm live larvae. T0 = day 0; T1 = day 11; T2 = day 22; T3 = day 33.

**FIGURE 3:**
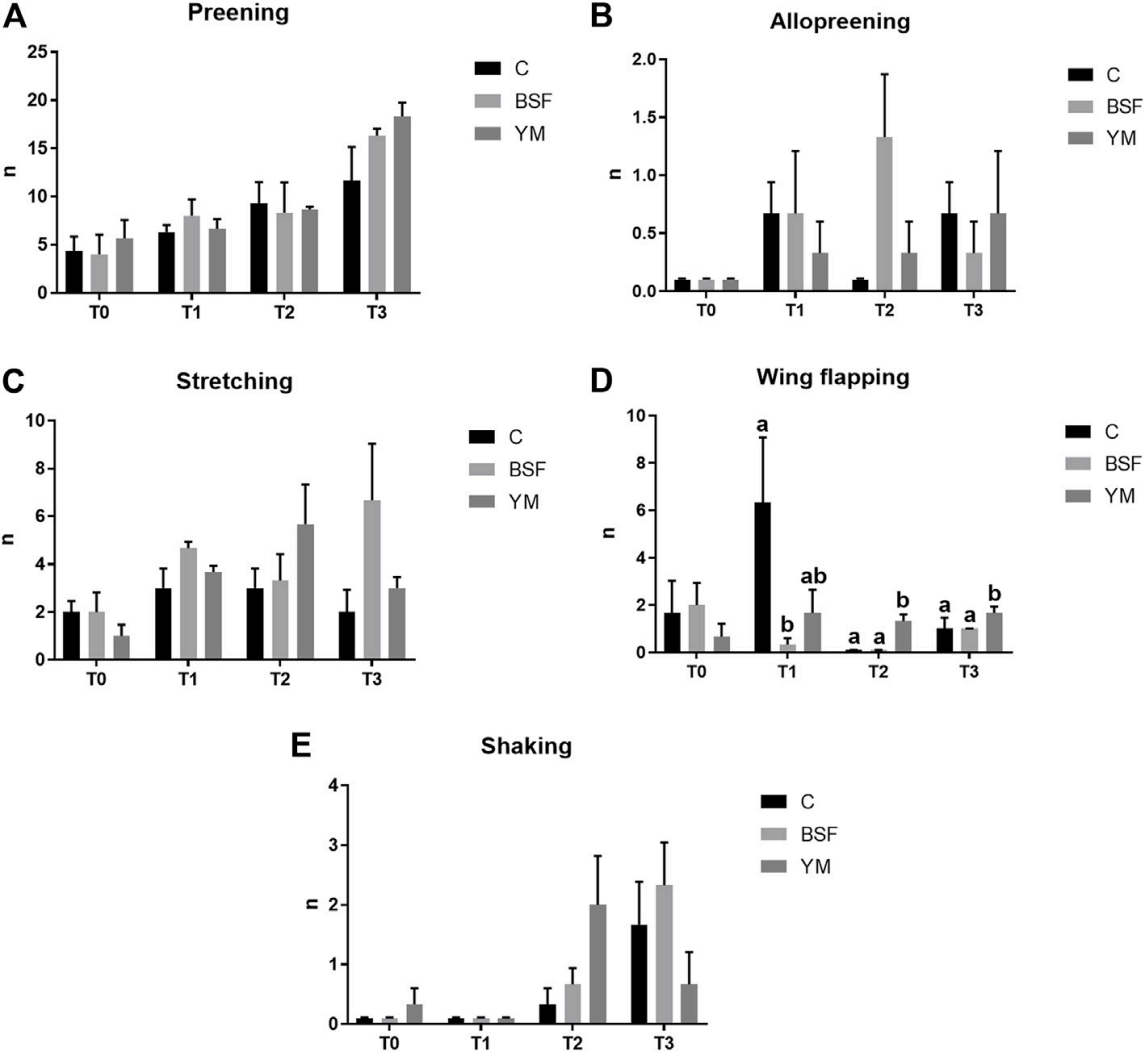
Frequency behaviours of the broiler chickens in the afternoon (diet*time interaction). **(A)** Preening. **(B)** Allopreening. **(C)** Stretching. **(D)** Wing flapping. **(E)** Shaking. Graph bars (representing least square means) with different superscript letters (a, b) indicate significant differences among the experimental treatments within the experimental times. C = control group; BSF = C diet + black soldier fly live larvae; YM = C diet + yellow mealworm live larvae. T0 = day 0; T1 = day 11; T2 = day 22; T3 = day 33.

Frequency behaviours of the broiler chickens of the present study are summarized in Table 3 and Figures 1–3. In the morning, stretching and wing flapping were influenced by both the insect live larvae administration and the time (*p* < 0.001), but no diet × time interaction was identified (*p* = 0.686 and *p* = 0.220, respectively). In details, the insect-fed broiler chickens performed more stretching and wing flapping than the C group (*p* < 0.001), and, independently of diet, a reduction (stretching) and an increase (wing flapping) of such behaviours was overall observed along the experimental trial (*p* < 0.001 and *p* = 0.010, respectively). The wing flapping frequency also abruptly decreased at T3 when compared to the other experimental times (*p* = 0.010). Preening depended on time only, with an increase being overall identified along the experimental trial, but an abrupt reduction at T3 (*p* = 0.001). On the contrary, no influence of insect live larvae administration or diet × time interaction were highlighted (*p* = 0.102 and *p* = 0.110, respectively). Allopreening, pecking pen mate and shaking behaviours did not depend on any of the considered variables (diet: *p* = 0.549, *p* = 1.000 and *p* = 0.001, respectively; time: *p* = 0.549, *p* = 0.290 and *p* = 0.100, respectively; diet × time: *p* = 0.404, *p* = 1.000 and *p* = 1.000, respectively). During the larvae intake, scratching and wing flapping behaviours were influenced by insect live larvae administration only (*p* = 0.025 and *p* < 0.001, respectively). In particular, the insect-fed broilers performed more scratching in comparison with the C birds (*p* = 0.025), while increased frequency in wing flapping was identified in the YM group only (*p* < 0.001). Differently, no influence of time (*p* = 0.070 or *p* = 0.661, respectively) or diet × time interaction (*p* = 0.662 and *p* = 0.508, respectively) were identified. Preening and stretching behaviours were influenced by either the insect live larvae administration or the time (*p* < 0.001). In particular, the insect-fed birds displayed less preening and stretching than the C broilers, with the YM group furtherly showing reduced stretching when compared to the BSF-fed birds (*p* < 0.001). Furthermore, independently of diet, preening and stretching frequencies progressively increased in the last 11 days of the experimental trial (*p* < 0.001). On the contrary, no diet × time interaction was highlighted (*p* = 0.057 and *p* = 0.104, respectively). Trotting and shaking behaviours depended on time only, with trotting frequency progressively decreasing in the last 11 days of the experimental trial (*p* < 0.001), and shaking displaying the opposite trend (*p* < 0.001). Differently, no influence of insect live larvae administration (*p* = 0.098 or *p* = 0.687, respectively) or diet × time interaction (*p* = 1.000 and *p* = 0.492, respectively) were identified. Allopreening and pecking pen mate behaviours did not depend on any of the considered variables (diet: *p* = 0.624 and *p* = 0.105, respectively; time: *p* = 1.000 and *p* = 0.624, respectively; diet × time: *p* = 1.000 and *p* = 1.000, respectively). In the afternoon, a diet × time interaction was observed for wing flapping only (*p* < 0.001). In details, the YM-fed broiler chickens performed more wing flapping than the other groups at T2 and T3 only (*p* < 0.001), while the C birds displayed higher wing flapping than the HI group at T1 (*p* < 0.05, Figure 3). On the contrary, preening, stretching and shaking behaviours depended on time only, with increasing frequencies being highlighted along the experimental trial (*p* < 0.001). On the contrary, no influence of insect live larvae administration (*p* = 0.770, *p* = 0.302 or *p* = 0.378, respectively) or diet × time interaction (*p* = 0.127, *p* = 0.106 and *p* = 0.052, respectively) were highlighted. Allopreening was not influenced by any of the considered variables (diet: *p* = 1.000; time: *p* = 0.527; diet × time: *p* = 0.527).

**TABLE 3:** Frequency behaviours of the broiler chickens depending on diet, time and their interaction.

	Diet (D)			Time (T)				SEM		*p*-value			Wald test		
	C	BSF	YM	T0	T1	T2	T3	D	T	D	T	D×T	D	T	D×T
Morning															
Scratching, n	<0.5 times of occurrence														
Preening, n	9.72	8.35	9.96	5.45^a^	8.05^b^	26.70^c^	6.44^a^	9.34	0.88	0.102	0.001	0.110	4.980	13.913	4.342
Allopreening, n	0.00	0.00	0.00	0.00	0.00	0.00	0.48	0.00	0.03	0.549	0.549	0.404	1.200	1.200	1.810
Trotting, n	<0.5 times of occurrence														
Stretching, n	2.07^a^	4.08^b^	4.74^b^	2.92^a^	2.89^ab^	3.31^ab^	4.91^b^	0.26	0.71	<0.001	<0.001	0.686	45.794	18.871	0.842
Pecking pen mate, n	0.00	0.00	0.00	0.00	0.00	0.67	0.00	0.00	0.17	1.000	0.290	1.000	0.000	2.412	0.000
Chasing, n	<0.5 times of occurrence														
Dust bathing, n	<0.5 times of occurrence														
Wing flapping, n	0.00^a^	1.67^b^	2.77^c^	1.88^b^	1.44^b^	6.38^a^	0.00^c^	0.21	0.25	<0.001	0.010	0.220	136.671	9.294	3.030
Shaking, n	0.00	0.00	0.00	0.00	0.00	0.87	0.00	0.00	0.03	1.000	0.100	1.000	0.000	4.280	0.000
During larvae intake															
Scratching, n	0.33^a^	2.28^b^	2.52^b^	1.20	1.21	1.06	1.49	0.27	0.41	0.025	0.070	0.662	7.416	9.787	0.825
Preening, n	13.05^a^	3.59^b^	4.74^b^	4.00^a^	3.85^a^	7.32^b^	7.89^b^	1.16	0.98	<0.001	<0.001	0.057	75.693	206.003	5.716
Allopreening, n	0.00	0.00	0.00	0.40	0.42	0.00	0.00	0.00	0.07	0.624	1.000	1.000	0.240	0.000	0.000
Trotting, n	0.00	0.00	0.00	1.31^a^	1.46^a^	0.00^b^	0.00^b^	0.00	0.13	0.098	<0.001	1.000	4.645	39.095	0.000
Stretching, n	4.89^a^	2.00^b^	1.39^c^	1.70^a^	1.88^a^	2.65^b^	2.71^b^	0.52	0.29	<0.001	<0.001	0.104	16.280	15.192	4.532
Pecking pen mate, n	0.00	0.00	0.53	0.00	0.00	0.00	0.00	0.07	0.00	0.105	0.624	1.000	4.950	0.786	0.000
Chasing, n	<0.5 times of occurrence														
Dust bathing, n	<0.5 times of occurrence														
Wing flapping, n	3.15^a^	2.63^a^	4.73^b^	3.45	3.61	3.86	2.81	0.31	0.79	<0.001	0.661	0.508	82.131	0.829	1.356
Shaking, n	0.00	0.00	1.01	0.00^a^	0.00^a^	0.00^a^	2.27^b^	0.22	0.03	0.687	<0.001	0.492	0.752	84.592	0.472
Afternoon															
Scratching, n	<0.5 times of occurrence														
Preening, n	7.39	8.12	8.80	4.61^a^	6.96^b^	8.77^c^	15.17^d^	1.15	0.90	0.770	<0.001	0.127	0.522	143571.734	4.125
Allopreening, n	0.00	0.00	0.00	0.00	0.53	0.00	0.53	0.00	0.17	1.000	0.527	0.527			
Trotting, n	<0.5 times of occurrence														
Stretching, n	3.73	5.46	4.26	1.59^a^	4.61^b^	6.31^b^	8.33^c^	0.66	0.52	0.302	<0.001	0.106	1.891	49.443	5.231
Pecking pen mate, n	<0.5 times of occurrence														
Chasing, n	<0.5 times of occurrence														
Dust bathing, n	<0.5 times of occurrence														
Wing flapping, n	0.00	0.00	1.25	1.30	1.52	0.00	1.19	0.09	0.31	0.309	0.888	0.001	2.346	0.237	14.554
Shaking, n	0.00	0.00	0.00	0.00^a^	0.00^a^	0.76^b^	1.37^c^	0.00	0.09	0.378	<0.001	0.052	1.947	20.694	5.975

C = control group; BSF = C diet + black soldier fly live larvae; YM = C diet + yellow mealworm live larvae. T0 = day 0; T1 = day 11; T2 = day 22; T3 = day 33. Means with superscript letters (a, b, c, d) denote significant differences (*p* < 0.05).

**FIGURE 4:**
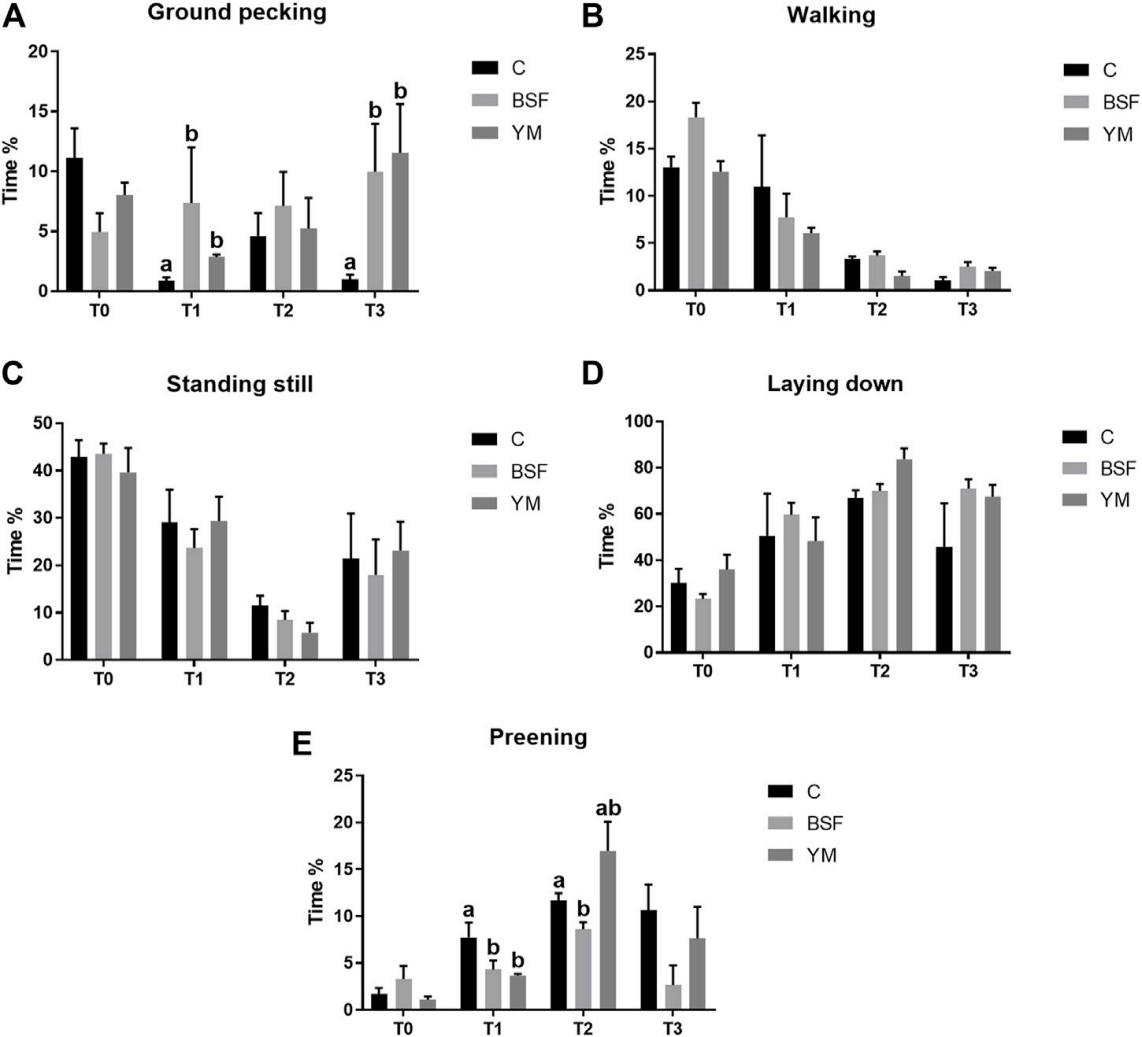
Duration behaviours of the broiler chickens in the morning (diet*time interaction). **(A)** Preening. **(B)** Allopreening. **(C)** Stretching. **(D)** Wing flapping. **(E)** Shaking. Graph bars (representing least square means) with different superscript letters (a, b) indicate significant differences among the experimental treatments within the experimental times. C = control group; BSF = C diet + black soldier fly live larvae; YM = C diet + yellow mealworm live larvae. T0 = day 0; T1 = day 11; T2 = day 22; T3 = day 33.

**FIGURE 5:**
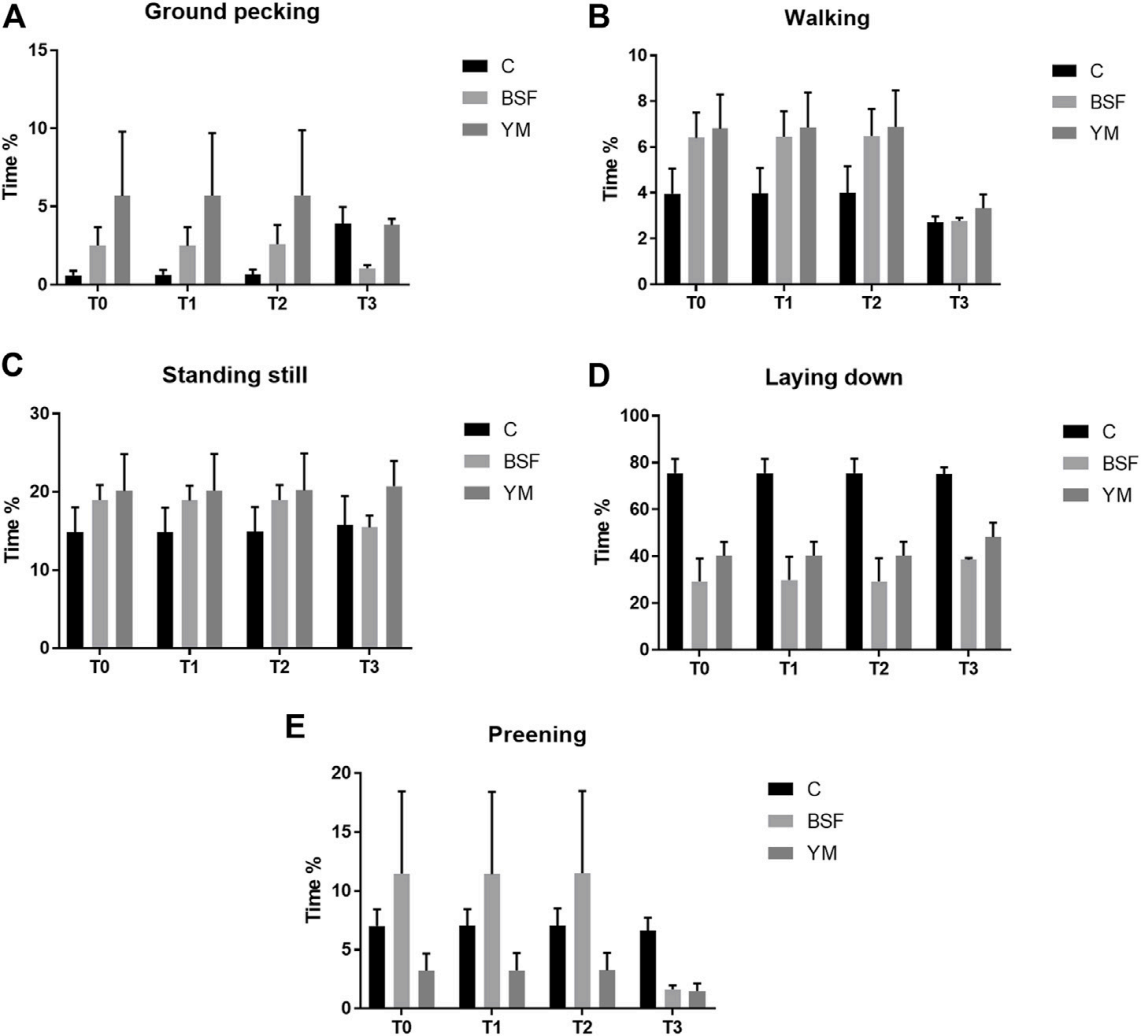
Duration behaviours of the broiler chickens during the larvae intake (diet*time interaction, *p* > 0.05). **(A)** Preening. **(B)** Allopreening. **(C)** Stretching. **(D)** Wing flapping. **(E)** Shaking. C = control group; BSF = C diet + black soldier fly live larvae; YM = C diet + yellow mealworm live larvae. T0 = day 0; T1 = day 11; T2 = day 22; T3 = day 33.

**FIGURE 6:**
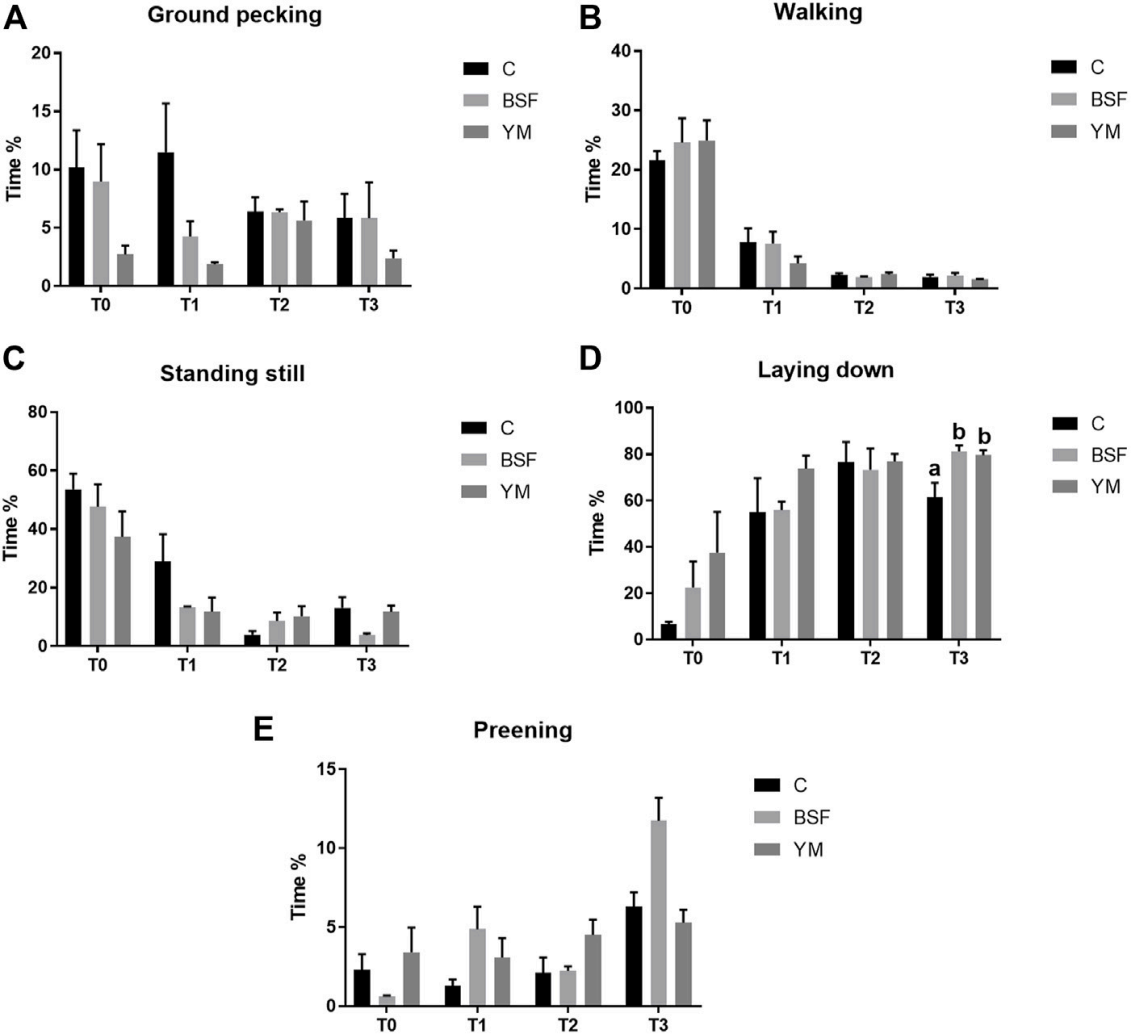
Duration behaviours of the broiler chickens in the afternoon (diet*time interaction). **(A)** Preening. **(B)** Allopreening. **(C)** Stretching. **(D)** Wing flapping. **(E)** Shaking. Graph bars (representing least square means) with different superscript letters (a, b) indicate significant differences among the experimental treatments within the experimental times. C = control group; BSF = C diet + black soldier fly live larvae; YM = C diet + yellow mealworm live larvae. T0 = day 0; T1 = day 11; T2 = day 22; T3 = day 33.

Duration behaviours of the broiler chickens of the current research are summarized in Table 4 and Figure 4–6. In the morning, a diet × time interaction was observed for both the ground pecking and the preening (*p* < 0.001 and *p* = 0.006, respectively). In particular, higher ground pecking was observed in the insect-fed broilers than the C group at T1 and T3 only (*p* < 0.001, Figure 4), whereas the C birds spent more time preening in comparison with the other groups or BSF group alone at T1 and T2, respectively (*p* = 0.006, Figure 4). Walking depended on either the insect live larvae administration or the time (*p* = 0.001 and *p* < 0.001, respectively). In details, the BSF birds spent more time walking when compared to the C group (*p* < 0.001), and, independently of diet, less walking was progressively observed along the experimental trial (*p* < 0.001). Differently, no diet × time interaction was identified (*p* = 0.186). Standing still and laying down behaviours were influenced by time only (*p* < 0.001 and *p* = 0.045, respectively). In particular, broiler chickens spent less time standing still along the experimental trial (*p* < 0.001), with an increase in laying down being also observed (*p* < 0.05). During the larvae intake, ground pecking and laying down depended on insect live larvae administration only (*p* < 0.001). In particular, the YM-fed birds displayed higher and lower, respectively, ground pecking and preening than the other groups, with either the BSF or the YM broilers spending less time laying down when compared to the C group (*p* < 0.001). On the contrary, no influence of time (*p* = 0.703 and *p* = 0.190, respectively) or diet × time interaction (*p* = 0.118 and *p* = 0.141, respectively) were highlighted. Preening was influenced by both the insect live larvae administration and the time (*p* < 0.001 and *p* = 0.001, respectively). In details, the YM-fed birds displayed lower preening than the other groups (*p* < 0.001), and, independently of diet, preening duration was reduced in the last 11 days of the experimental trial (*p* = 0.001). Differently, no diet×time interaction was identified (*p* = 0.060). On the contrary, no influence of insect live larvae administration or diet × time interaction were observed (*p* = 0.208 and *p* = 0.077, respectively). Standing still did not depend on any of the considered variables (diet: *p* = 0.218; time: *p* = 0.710; diet × time: *p* = 0.058). In the afternoon, the insect-fed birds showed higher laying down in comparison with the C group at T3 only (diet × time interaction, *p* < 0.001; Figure 6). Ground pecking behaviour depended on insect live larvae administration, with the YM-fed broiler chickens spending less time ground pecking than the other groups (*p* < 0.001). On the contrary, no influence of time or diet × time interaction were highlighted (*p* = 0.110 and *p* = 0.571, respectively). Finally, walking, standing still and preening behaviours were influenced by time only (*p* < 0.001), with broiler chickens spending less time walking and standing still, as well as more time preening, along the experimental trial (*p* < 0.001). Differently, no influence of insect live larvae administration (*p* = 0.678, *p* = 0.414 and *p* = 0.285, respectively) or diet × time interaction (*p* = 0.112, *p* = 0.215 and *p* = 0.116, respectively) were observed.

**TABLE 4:** Duration behaviours of the broiler chickens depending on diet, time and their interaction.

	Diet (D)			Time (T)				SEM		*p*-value			Wald test		
	C	BSF	YM	T0	T1	T2	T3	D	T	D	T	D×T	D	T	D×T
Morning															
Ground pecking, time %	2.59^a^	7.12^ab^	6.11^b^	7.62^a^	2.64^c^	5.55^b^	4.88^b^	0.89	0.49	<0.001	<0.001	<0.001	101.932	366.984	235.8011
Walking, time %	4.74^a^	5.99^b^	3.95^ab^	14.43^a^	8.00^b^	2.76^b^	1.66^c^	0.45	0.88	0.001	<0.001	0.186	14.706	128.630	3.362
Standing still, time %	23.52	19.91	19.89	41.98^a^	27.22^b^	8.28^c^	20.71^b^	2.67	3.21	0.573	<0.001	0.355	1.115	37.646	2.070
Laying down, time %	46.45	51.23	56.03	29.36^a^	52.59^ab^	73.21^b^	60.24^b^	3.49	6.56	0.055	0.045	0.107	5.793	6.184	16.710
Preening, time %	7.91^a^	4.72^b^	7.34^b^	2.02^a^	5.24^b^	12.40^d^	6.98^c^	0.84	0.88	0.019	0.004	0.006	7.906	11.024	10.203
During larvae intake															
Ground pecking, time %	1.61^ab^	1.66^a^	2.52^b^	2.10	2.06	2.14	2.52	0.85	0.58	<0.001	0.703	0.118	93.006	0.146	5.674
Walking, time %	3.29	4.24	4.78	5.58^a^	5.50^a^	5.64^a^	2.92^b^	0.63	0.24	0.208	<0.001	0.077	3.139	38.806	5.132
Standing still, time %	15.32	17.12	20.45	18.58	18.20	17.86	17.15	1.93	1.53	0.218	0.710	0.058	3.050	0.139	6.008
Laying down, time %	75.27^a^	33.65^b^	44.08^b^	43.39	42.78	44.69	51.88	4.55	2.84	<0.001	0.190	0.141	251.827	1.714	3.918
Preening, time %	6.82^a^	4.33^a^	2.20^b^	5.75^a^	5.90^a^	6.40^a^	2.53^b^	1.01	0.66	<0.001	0.001	0.060	140.920	12.020	5.640
Afternoon															
Ground pecking, time %	8.12^a^	6.13^a^	2.87^b^	6.26	4.51	6.09	4.34	1.00	0.85	<0.001	0.110	0.571	19.931	4.421	1.120
Walking, time %	5.17	5.25	4.42	23.65^a^	6.18^b^	2.17^c^	1.86^d^	0.65	1.03	0.678	<0.001	0.112	0.778	18619.759	4.980
Standing still, time %	16.85	14.79	15.15	45.74^a^	16.45^b^	7.05^c^	11.08^b^	1.95	1.64	0.414	<0.001	0.215	1.761	1013.777	3.165
Laying down, time %	36.04^a^	52.31^b^	64.22^b^	17.65^a^	60.99^b^	75.61^b^	73.60^b^	5.55	5.09	<0.001	<0.001	<0.001	370.193	44.580	486.225
Preening, time %	2.50	2.96	3.97	1.67^a^	2.67^b^	2.78^b^	7.32^c^	0.42	0.47	0.285	<0.001	0.116	2.510	11294.008	5.125

C = control group; BSF = C diet + black soldier fly live larvae; YM = C diet + yellow mealworm live larvae. T0 = day 0; T1 = day 11; T2 = day 22; T3 = day 33. Means with superscript letters (a, b, c, d) denote significant differences (*p* < 0.05).

### Feet and hock health assessment

The administration of BSF and YM live larvae did not influence either the HB (H = 3.644; C: 0.37 ± 0.09; BSF: 0.73 ± 0.15; YM: 0.77 ± 0.17) or the FPD (H = 2.603; C: 0.60 ± 0.15; BSF: 0.60 ± 0.14; YM: 0.33 ± 0.11) scores (*p* = 0.162 and *p* = 0.272, respectively).

### Excreta corticosterone

**FIGURE 7:**
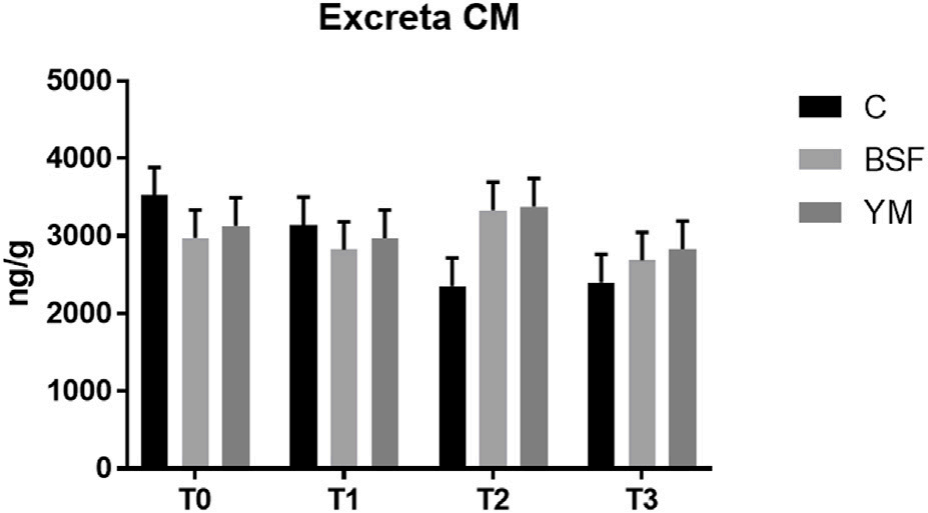
Excreta CM of the broiler chickens (diet*time interaction, *p* > 0.05). C = control group; BSF = C diet + black soldier fly live larvae; YM = C diet + yellow mealworm live larvae. T0 = day 0; T1 = day 11; T2 = day 22; T3 = day 33.

The excreta CM of the broiler chickens of the present study are summarized in Table 5 and Figure 7. The administration of BSF and YM live larvae did not affect the excreta CM of the broiler chickens of the current research (*p* = 0.684). Similarly, no time-related effects or diet × time interactions were identified (*p* = 0.288 and *p* = 0.369, respectively).

**TABLE 5:** Excreta CM of the broiler chickens depending on diet, time and their interaction.

	Diet (D)			Time (T)				SEM		*p*-value			Wald test		
	C	BSF	YM	T0	T1	T2	T3	D	T	D	T	D×T	D	T	D×T
CM, ng/g	2855.8	2955.6	3079.4	3210.3	2978.2	3024.4	2641.4	181.1	209.2	0.684	0.288	0.369	0.382	1.284	1.108

C = control group; BSF = C diet + black soldier fly live larvae; YM = C diet + yellow mealworm live larvae. T0 = day 0; T1 = day 11; T2 = day 22; T3 = day 33.

## Discussion

### Feathering score

The administration of neither the BSF nor the YM live larvae was able to improve the feathering scores of the broiler chickens of the present study. Previous research highlighted a tendency towards improvement or a significant improvement in feather damage of BSF live larvae-fed turkey poults and laying hens, respectively (Veldkamp and van Niekerk, 2019; Star et al., 2020). Such improvement has been related to a reduction in the aggressive pecking directed at the back and tail base, as a consequence of the re-direction of this behaviour towards the floor and away from feathers (Veldkamp and van Niekerk, 2019). However, since the aggressive pecking displayed by the broilers of the current research was not influenced by the administration of either the BSF or the YM live larvae, it is reasonable that feather conditions were unaffected as well. Independently of the utilization of the insect larvae, the back and the wing of the birds showed the best and the worst feather coverage, respectively. Little information is currently available on the feathering scores of the different body parts in broiler chickens (Lai et al., 2010; Mahmoud et al., 2015; Sevim et al., 2022), with the totality of the body areas being not always assessed (Sevim et al., 2022), or the authors reporting a mean body score only (Mahmoud et al., 2015). Lai et al. (2010) previously identified similar feathering scores among the different body regions of broiler chickens, while a clear separation between the back and the other body areas was herein outlined. The poor feather coverage of the breast can reasonably be attributed to the clear predominance of laying down behaviour in the whole behavioural time budget of the birds, while wing, under-wing and tail feather damage may be related to the progressively increase in preening frequency and duration along the experimental trial. Indeed, wing and tail—along with breast—represent the plumage areas receiving preferred attention from the birds during preening (Duncan and Wood-Gush, 1972). A significant role of the genetic selection—which aims at growth of meat and not feathers—cannot be excluded as well.

### Behaviour analysis

The variations in the behavioural repertoire of the broiler chickens of the present study share several similarities between the morning and the moment of the larvae intake, while the afternoon was characterized by different behavioural patterns. During the morning and the larvae intake, birds receiving the insect live larvae spent more time ground pecking (with a statistical significance being detected at T1 and T3 only, as a consequence of the higher SEM of T2) and performing increased scratching behaviour when compared to the non-supplemented animals. This clear stimulation of a more natural behaviour such as foraging [characterized by ground pecking and/or scratching (Ipema et al., 2020)] has already been observed in turkey poults and broiler chickens administered with BSF live larvae (Veldkamp and van Niekerk, 2019; Ipema et al., 2020). Scattering food items on the litter (such insects) or using different bedding materials (sand, moss-peat, or oat husks) have previously been reported to stimulate foraging behaviour in broiler chickens (Arnould et al., 2004; Baxter and O’Connell, 2016; Pichova et al., 2016). However, similar environmental enrichments (such as whole wheat, wood shavings, rice hulls or straw pellets) are not capable of exerting an analogous effect (Bizeray et al., 2002; Shields et al., 2005; Toghyani et al., 2010; Jordan et al., 2011; Baxter and O’Connell, 2016; Pichova et al., 2016), thus suggesting that birds have a clear preference for certain types of substrates (Riber et al., 2018). Indeed, the motivational significance behind each food-based enrichment represents the main driver of the behavioural changes (Pichova et al., 2016), and the insect larvae—as alive, moving and part of the natural diet of birds—seem to be highly interesting for poultry (Bokkers and Koene, 2002; Bruce et al., 2003; Ipema et al., 2020). The same motivational significance reasonably determined the increase in the activity levels of the insect-fed broiler chickens of the current research as well, as demonstrated by the increased frequency of stretching and wing flapping behaviours (the latter being mainly detected in the YM-fed birds), the increased time spent for walking and performing wing flapping, and the decreased time spent for laying down. An analogous scenario was also underlined in broilers and laying hens administered with BSF or YM live larvae as environmental enrichment (Pichova et al., 2016; Ipema et al., 2020; Star et al., 2020). It is, however, interesting to notice that the increase in stretching was observed in the morning only, while during the larvae intake such behaviour actually decreased. This may reasonably be related to the parallel increase in scratching and wing flapping behaviours. Another peculiar aspect to highlight is the reduced frequency (independently of time) and duration (mainly with BSF, as a consequence of the higher SEM of the YM group) preening displayed by the insect-fed birds of the present study. Preening, as it keeps plumages well-groomed by distributing lipid-rich oils from uropygial glands and removing parasites (Delius, 1988), could take a large time budget (∼13%) out of the total behaviour repertoire of domestic fowl (Dawkins, 1989). However, overall time spent preening and number of preening bouts could give useful information about environment appropriateness for birds (Li et al., 2020). Indeed, absence of environmental stimuli (i.e., cages) stimulates the birds to spend more time preening (Delius, 1988) or to perform short-term and frequent preening (Duncan, 1998), as a sign of boredom and frustration. Therefore, the administration of insect live larvae may reduce such negative feelings in broilers. In the afternoon, birds receiving YM live larvae spent less time ground pecking than the other groups, whereas either the BSF- or the YM-fed broilers showed an increased duration of laying down behaviour (with a statistical significance being detected at T3 only, as a consequence of the higher SEM of T1 and T2). This may indicate that the need for foraging was fully rewarded during the morning and the larvae intake, and that the overall increased activity observed in the first part of the day predisposed the birds to rest in the afternoon. However, the wing flapping frequency remained higher in the YM-fed broiler chickens when compared to the other groups (with a statistical significance being detected in the last third of the experimental trial only, as a consequence of the higher SEM of T1).

Independently of the administration of the insect live larvae, the broiler chickens of the present study displayed less active behaviours (i.e., ground pecking, walking and standing still), as well as more passivity (i.e., laying down), with increasing age. This is in agreement with previous research on broilers (Bokkers and Koene, 2003; Castellini et al., 2016; Ipema et al., 2020; Jacobs et al., 2021), where the rapid increase in body weights leads to poor mobility and, in turn, inhibits their ability to express certain behaviours (Bokkers, 2004; Castellini et al., 2016). The overall increase in preening may similarly be attributed to frustration related to poor mobility (Bokkers and Koene, 2003). On the contrary, other active behaviours such as stretching, shaking and wing flapping increased with increasing age of birds. It is, however, important to underline that fast-growing broilers are motivated to perform the normal behavioural repertoire of chickens, even after 6 weeks of age and despite being hampered by the high body weights (Bokkers, 2004). Furthermore, as behaviours are performed in sitting position rather than in standing position with increasing age (Bokkers, 2004), it is reasonable to identify an increase in behaviours that birds can easily perform when laying down.

As a final aspect to consider, the use of YM live larvae yielded slightly more pronounced effects on bird behaviour (especially in terms of stimulation of foraging and increase in activity levels) than the BSF ones. Considering that the broiler chickens of the current research spent less time consuming the YM live larvae when compared to BSF (Bellezza Oddon et al., 2021), it is possible to speculate a bird preference towards the larvae of this insect species. However, further studies are needed to confirm this hypothesis.

### Feet and hock health assessment

Similarly to what was observed for the feathering scores, the HB and the FPD scores of the broiler chickens of the current research were not influenced by the administration of either the BSF or the YM live larvae. Ipema et al. (2020) highlighted that FPD occurrence was not affected by insect live larvae provision, whereas the larvae-administered birds displayed less HB when compared to the C birds. However, considering that FPD incidence has been reported to be influenced only in the first 3 weeks of age in turkey poults (Veldkamp and van Niekerk, 2019), it is reasonable that a single evaluation may not be enough to observe potential differences in broilers as well. Furthermore, the identification of very low mean values for both the HB and the FPD scores of the C birds (less than 1) suggested the presence of an health status of the legs that was already good independently of insect live larvae administration, thus, in turn, making more challenging to improve it.

### Excreta corticosterone

The excreta CM of the broiler chickens of the present study were not affected by the administration of both the BSF and the YM live larvae as well. The measurement of excreta CM is a well-recognized, non-invasive method to quantify the stress response in poultry, which offers a more convenient and less disruptive alternative to traditional measures that require bird restraint and blood sampling (Weimer et al., 2018), and does not interrupt the animal behaviour (Hirschenhauser et al., 2012). However, it is fundamental to underline that many factors (such as age, sex, diet, metabolic rate, social status, early life experience, diurnal and seasonal variations, and differences in the hormone metabolism of individuals) may influence the excreta CM (Alm et al., 2014). Therefore, despite the positive, insect-related modulation in the bird behaviour herein highlighted, such variability could have probably hidden the potential differences in the excreta CM.

## Conclusion

In conclusion, the administration of BSF and YM live larvae as environmental enrichment for broiler chickens was capable of positively influencing the bird welfare through the stimulation of foraging behaviour, increase in activity levels, and reduction of behaviours potentially attributable to frustration, without affecting the plumage status, the leg health, and the excreta CM. As behavioural outcomes suggested some preference of the broilers for YM live larvae, further research to confirm this preference is recommended. Considering that the administration of insect live larvae in the intensive farming may potentially lead to different outcomes—as a consequence of the high rearing densities and competitiveness among birds—additional research testing such innovative environmental enrichment in the commercial setup are strongly recommended.

## Data Availability

The raw data supporting the conclusions of this article will be made available by the corresponding author upon reasonable request.
